# Removal of a Portion of the Teat of a Cow*Wochenschrift für Thierheilkunde und Viehsucht.

**Published:** 1885-01

**Authors:** 


### II.—REMOVAL OF A PORTION OF THE TEAT OF A COW.*

A cow laboring under difficulty of milking was obliged to wear a rubber tube without a button at the end, which passed through the entire duct of the teat. Another tube was introduced which pushed the first to the extremity. I was called for the purpose of extracting the tube, which was done after much labor with a fine tongs, by means of which I drew out the first portion by a twisting movement. To extract the upper portion I was obliged to remove a considerable part of the teat. Strange to say, contrary to what I expected after the healing of the wound, the secretion of the milk proceeded as well as ever, and discharged itself through the stump whilst the other teats were being milked. I saw the animal again after the following delivery, and found everything concerned in the milk secretion doing well.

* Wochenschrift für Thierheilkunde und Viehsucht.

